# Supplementary material for: Phylogenetic Analysis of Escherichia coli Isolated from Australian Feedlot Cattle in Comparison to Pig Faecal and Poultry/Human Extraintestinal Isolates
Source: Antibiotics (Basel). 2023 May 11;12(5):895. doi: 10.3390/antibiotics12050895 (PMC10215834; doi:10.3390/antibiotics12050895)
Supplement: Supplementary file 1 [file antibiotics-12-00895-s001.zip › antibiotics-2360019-supplementary.pdf]

Supplementary Materials

Table S1. Prevalence of antimicrobial resistance genes, pMLST, and Class 1 integrase gene in *E. coli* isolated from beef cattle, pig, poultry, and human.

Sample ID	Source	ESBL Production	Phylogroup	MLST	Antimicrobial Resistance Genes	pMLST	Integron
19MLAN001	Beef cattle	No	A	10		F2:A-:B-	
19MLAN002	Beef cattle	No	B1	297		F57:A-:B43	
19MLAN054	Beef cattle	No	C	88	*aph(3')-Ia, aph(3'')-Ib, aph(6)-Id, bla*$_{TEM-1B}$*, dfrA5,sul2*	F2:A-:B1	*intI1*
19MLAN112	Beef cattle	Yes	A	1102	*bla*$_{CMY-2}$*, bla*$_{TEM-1B}$*, dfrA5, tet(A),*	F40:A-:B16	*intI1*
19MLAN305	Beef cattle	No	A	10	*tet(B)*	F89:A-:B-	
19MLAN307	Beef cattle	Yes	A	10	*aph(3'')-Ib, aph(6)-Id, bla*$_{CTX-M-27}$*, mph(A), sul2, tet(A)*	F2:A-:B-	
19MLAN308	Beef cattle	No	B1	1665		F40:A-:B-	
19MLAN309	Beef cattle	No	B1	2602		F34:A-:B16	
19MLAN314	Beef cattle	No	A	10	*tet(B)*	F89:A-:B-	
19MLAN316	Beef cattle	No	B1	278	*tet(B)*		
19MLAN321	Beef cattle	No	A	1122	*bla*$_{TEM-1C}$*, tet(A)*	F-:A-:B38	
19MLAN324	Beef cattle	No	A	10	*aph(3'')-Ib, aph(6)-Id, bla*$_{TEM-1B}$*, tet(B),*	F104:A-:B16	
19MLAN325	Beef cattle	Yes	B1	1494	*bla*$_{CTX-M-15}$*, mph(E), mph(A), msr(E), tet(A)*	F40:A-:B-	
19MLAN330	Beef cattle	Yes	E	3381	*bla*$_{CMY-2}$	F57:A-:B16	
19MLAN333	Beef cattle	No	A	10	*aph(3'')-Ib, aph(6)-Id, bla*$_{TEM-1B}$, tet(B)	F104:A-:B16	
19MLAN338	Beef cattle	No	A	2035	*sul2*		
19MLAN341	Beef cattle	No	A	10	*aph(3'')-Ib, aph(6)-Id, bla*$_{TEM-1B}$*, tet(B)*	F104:A-:B16	
19MLAN344	Beef cattle	No	B1	515	*bla*$_{TEM-1B}$	F18:A5:B1	
19MLAN347	Beef cattle	Yes	A	10	*aph(3'')-Ib, aph(6)-Id, bla*$_{CTX-M-27}$*, sul2, mph(A), tet(A)*	F2:A-:B-	
19MLAN348	Beef cattle	No	B1	515	*tet(A)*	F89:A-:B-	
19MLAN351	Beef cattle	No	B1	278	*tet(B)*		
19MLAN352	Beef cattle	No	B1	278	*tet(B)*		
19MLAN354	Beef cattle	Yes	B1	9967	*aac(3)-IV, aph(4)-Ia, aph(3'')-Ib, aph(6)-Id, ant(3'')-Ia, bla*$_{CTX-M-15}$*, bla*$_{TEM-1B}$*, cmlA1, dfrA12 , qnrS1, sul1,* sul3, *tet(A)*		*intI1*
19MLAN355	Beef cattle	No	B1	641	*tet(A)*	F95:A-:B-	
19MLAN358	Beef cattle	No	B1	223	*bla*$_{TEM-1C}$*, tet(A)*	F89:A-:B-	
19MLAN369	Beef cattle	No	B1	109	*aph(3'')-Ib, aph(6)-Id, sul2, tet(B)*	F95:A-:B-	
19MLAN373	Beef cattle	No	B1	109	*aph(3'')-Ib, aph(6)-Id, sul2, tet(B)*	F95:A-:B-	
19MLAN381	Beef cattle	No	A	10	*aph(3'')-Ib, aph(6)-Id, bla*$_{TEM-1B}$*, tet(B)*	F-:A-:B16	
19MLAN385	Beef cattle	No	A	2035	*sul2, tet(B)*		
19MLAN396	Beef cattle	No	A	10		F-:A-:B57	
19MLAN401	Beef cattle	No	A	278	*tet(B)*		
19MLAN413	Beef cattle	No	B1	278	*tet(B)*		
19MLAN415	Beef cattle	No	B1	641	*tet(A)*	F89:A-:B-	
19MLAN423	Beef cattle	Yes	A	540	*aph(3'')-Ib, aph(6)-Id, bla*$_{CTX-M-15}$*, bla*$_{TEM-1B}$*, dfrA14, sul2, tet(A)*	F-:A-:B53	*intI1*
19MLAN435	Beef cattle	No	B1	278	*tet(B)*		
19MLAN442	Beef cattle	No	B1	278	*tet(A)*		
19MLAN443	Beef cattle	No	B1	58	*aph(3'')-Ib, aph(6)-Id, sul2, tet(B)*		
SRR11658524	Pig	NA	A	744	*aadA1, aadA2, aadA5, aph(3')-Ia, aph(3'')-Ib, aph(6)-Id, bla*$_{TEM-1B}$*, catA1, cmlA1, dfrA12, sul1, gyrA, mef(B), mph(A), tet(A), tet(B)*	F10:A6:B42	*intI1*
SRR11658525	Pig	NA	A	8166	*aadA1, aadA2, bla*$_{TEM-1B}$*, cmlA1, dfrA12, sul3,*	F-:A-:B-	*intI1*
SRR11658528	Pig	NA	A	6615	*aadA1, aadA2, bla*$_{TEM-1B}$*, cmlA1, dfrA12, sul3, tet(A)*	F-:A-:B-	*intI1*
SRR11658531	Pig	NA	B1	877	*bla*$_{TEM-1B}$*, dfrA5,*	F34:A-:B-	*intI1*
SRR11658533	Pig	NA	A	34	*aph(3')-Ia, aph(3'')-Ib, aph(6)-Id, bla*$_{TEM-1B}$*, catA1, dfrA5,sul2, tet(A), tet(B)*	F55:A-:B1	*intI1*

SRR11658536	Pig	NA	A	8167	aadA1, aadA2, aph(3'')-Ib, aph(6)-Id, bla$_{TEM-1B}$, cmlA1, dfrA12, mef(B), sul2, tet(A), tet(B)	F-:A-:B-	intI1
SRR11658538	Pig	NA	A	361	aadA1, aadA2, aph(3')-Ia, bla$_{TEM-1B}$, cmlA1, dfrA12, gyrA, mef(B), parC, sul3, tet(A)	F-:A18:B-	intI1
SRR11658548	Pig	NA	A	48	aadA1, aadA2, blaCARB-2, cmlA1, dfrA16, gyrA, sul3, tet(A)	F-:A-:B-	intI1
SRR11658549	Pig	NA	B1	641	bla$_{TEM-1B}$, dfrA5, sul1	F2:A-:B1	intI1
SRR11658550	Pig	NA	B1	20	aph(3'')-Ib, aph(6)-Id, bla$_{TEM-1B}$, dfrA5, sul2, tet(A)	F115:A-:B42	intI1
SRR11658553	Pig	NA	B1	29	aac(3)-Iia, aadA1, aph(3'')-Ib, aph(6)-Id, bla$_{TEM-1B}$, dfrA5, sul1, sul2	F4:A-:B40	intI1
SRR11658554	Pig	NA	B1	11081	aadA2, aph(3'')-Ib, aph(6)-Id, bla$_{TEM-1B}$, dfrA12, sul1, tet(B)	F-:A-:B-	intI1
SRR11658555	Pig	NA	A	398	aadA1, aadA2, blaTEM-1B, cmlA1, dfrA5, dfrA12, sul3	F2:A-:B1	intI1
SRR11658556	Pig	NA	A	761	aac(2')-Iia, aadA1, aadA2, bla$_{TEM-1B}$, cmlA1, dfrA12, sul3, tet(B)	F-:A-:B-	intI1
SRR11658557	Pig	NA	A	744	aadA1, aadA2, aph(3'')-Ib, aph(6)-Id, bla$_{TEM-1B}$, cmlA1, dfrA12, gyrA, parC, sul2, sul3, tet(A)	F56:A-:B20	intI1
SRR11658558	Pig	NA	A	761	aac(2')-Iia, aadA1, aadA2, bla$_{TEM-1B}$, cmlA1, dfrA12, sul3, tet(B)	F-:A-:B-	intI1
SRR11658560	Pig	NA	A	361	aadA1, aadA2, aadA8, aph(3')-Ia, bla$_{TEM-1B}$, cmlA1, dfrA12, gyrA, parC, mef(B), sul3	F-:A18:B-	intI1
SRR11658561	Pig	NA	E	8868	aadA1, aadA2, aph(3')-Iia, aph(3'')-Ib, aph(6)-Id, bla$_{TEM-1B}$, cmlA1, dfrA12, sul1, sul3, tet(A)	F40:A-:B71	intI1
SRR11658564	Pig	NA	A	10	aac(3)-IV, aph(3')-Ia, aph(3'')-Ib, aph(6)-Id, bla$_{TEM-1B}$, dfrA5, sul2	F100:A-:B1	intI1
SRR11658565	Pig	NA	A	4628	aph(3'')-Ib, bla$_{TEM-1B}$, dfrA5, tet(A)	F4:A-:B-	intI1
SRR11658569	Pig	NA	A	48	aph(3'')-Ib, aph(6)-Id, bla$_{TEM-1B}$, dfrA14, sul2, tet(B)	F-:A-:B-	intI1
SRR11658570	Pig	NA	A	609	aadA1, aph(3'')-Ib, bla$_{TEM-1B}$, dfrA5	F2:A-:B1	intI1
SRR11658573	Pig	NA	B1	58	aph(3')-Ia, aph(3'')-Ib, aph(6)-Id, bla$_{TEM-1B}$, dfrA5, sul2	F2:A-:B1	intI1
SRR11658574	Pig	NA	A	Unknown	aph(3'')-Ib, aph(6)-Id, bla$_{TEM-1B}$, dfrA5, sul2, tet(A), tet(B)	F4:A2:B53	intI1
SRR11658577	Pig	NA	B1	154	aph(3'')-Ib, aph(6)-Id, bla$_{TEM-1B}$, dfrA5, sul2, tet(A)	F4:A6:B54	intI1
SRR11658579	Pig	NA	B1	Unknown	aadA2, aph(3'')-Ib, aph(6)-Id, bla$_{TEM-1B}$, dfrA12, tet(B)	F-:A-:B-	intI1
SRR11658587	Pig	NA	A	165	aph(3')-Ia, aph(3'')-Ib, aph(6)-Id, bla$_{TEM-1B}$, dfrA5, sul1, sul2, tet(B)	F-:A-:B-	intI1
SRR11658588	Pig	NA	A	202	aadA1, aadA2, bla$_{TEM-1B}$, cmlA1, dfrA12, sul3, tet(B)	F-:A-:B-	intI1
SRR11658589	Pig	NA	A	398	aadA1, bla$_{TEM-1B}$, cmlA1, dfrA5, dfrA12, sul3	F2:A-:B1	intI1
SRR11658591	Pig	NA	A	48	aph(3'')-Ib, aph(6)-Id, bla$_{TEM-1B}$, dfrA14, sul2, tet(B)	F-:A-:B-	intI1
SRR11658595	Pig	NA	A	542	aadA1	F-:A-:B-	intI1
SRR11658599	Pig	NA	A	4429	aadA1, aadA2, bla$_{TEM-1B}$, bla$_{TEM-206}$, bla$_{TEM-214}$, cmlA1, dfrA12, tet(A)	F-:A-:B-	intI1
SRR11658600	Pig	NA	A	398	aac(3)-IV, aph(3'')-Ib, bla$_{TEM-1B}$, bla$_{TEM-141}$, bla$_{TEM-206}$, bla$_{TEM-209}$, bla$_{TEM-214}$, bla$_{TEM-216}$, cmlA1, dfrA5, sul3	F-:A-:B-	intI1
SRR11658602	Pig	NA	B1	20	aph(3'')-Ib, aph(6)-Id, bla$_{TEM-1B}$, dfrA5, sul2, tet(A)	F-:A-:B42	intI1
SRR11658605	Pig	NA	A	8170	aadA1, aadA2, aph(3'')-Ib, aph(6)-Id, dfrA5, dfrA12, sul3, tet(B)	F56:A-:B-	intI1

SRR11658607	Pig	NA	B1	101	aph(3'')-Ib, aph(6)-Id, bla$_{TEM-1B}$, dfrA5, sul2, tet(A)	F4:A2:B16	intI1
SRR11658611	Pig	NA	B1	345	aph(3'')-Ib,aph(6)-Id, bla$_{TEM-1B}$,dfrA5, sul2, tet(A)	F4:A-:B1	intI1
SRR11658612	Pig	NA	B1	877	aph(3')-Ia, aph(3'')-Ib, aph(6)-Id, bla$_{TEM-1B}$, dfrA5, sul2, tet(A)	F56:A-:B20	intI1
SRR11658617	Pig	NA	A	361	aadA1, aadA2, aph(3')-Ia, bla$_{TEM-1B}$, cmlA1, dfrA12, gyrA, parC, mef(B), sul3, tet(A)	F-:A18:B-	intI1
SRR11658618	Pig	NA	A	10	aadA1, aadA2, bla$_{TEM-1B}$, cmlA1, dfrA12, sul3	F-:A-:B-	intI1
SRR11658627	Pig	NA	B1	101	aadA1, aadA2, aph(3'')-Ib, aph(6)-Id, bla$_{TEM-1B}$, cmlA1, dfrA5, dfrA12, sul2, sul3, tet(A)	F18:A-:B-	intI1
SRR11658635	Pig	NA	B1	8168	aadA1, aadA2, bla$_{TEM-1B}$, cmlA1, dfrA12, qnrS1, sul3, tet(B)	F-:A8:B-	intI1
SRR11658636	Pig	NA	A	361	aadA1, aadA2, aph(3')-Ia, bla$_{TEM-1B}$, cmlA1, dfrA12, gyrA, mef(B), parC, sul2, tet(A)	F-:A18:B-	intI1
SRR11658637	Pig	NA	A	226	aadA1, aadA2, bla$_{TEM-1B}$, cmlA1, dfrA12, sul3, tet(B)	F-:A-:B-	intI1
SRR11658639	Pig	NA	D	69	aph(3'')-Ib, aph(6)-Id, bla$_{TEM-1B}$, dfrA7, sul2	F52:A-:B48	intI1
SRR7469840	Poultry	NA	B2	95	aadA1, aph(3'')-Ib, aph(6)-Id, bla$_{TEM-1A}$, dfrA1, sul1, tet(A)	F24:A-:B1	intI1
SRR7469841	Poultry	NA	D	973	aph(3'')-Ib, aph(6)-Id, bla$_{TEM-1B}$, bla$_{TEM-206}$, bla$_{TEM-214}$, dfrA5, sul1, sul2, tet(A)	F24:A-:B8	intI1
SRR7469843	Poultry	NA	G	117	dfrA5, sul1, tet (C)	F18:A-:B1	intI1
SRR7469859	Poultry	NA	E	57	dfrA5, tet(A)	F2:A-:B1	intI1
SRR7469862	Poultry	NA	B1	155	bla$_{TEM-1B}$, dfrA5, sul1, tet(A)	F24:A-:B1	intI1
SRR7469865	Poultry	NA	D	973	aph(3'')-Ib, aph(6)-Id, dfrA5, sul1, sul2, tet(A)	F24:A-:B8	intI1
SRR7469866	Poultry	NA	D	8398	tet(C)	F82:A-:B1	intI1
SRR7469869	Poultry	NA	G	117	aph(3'')-Ib, aph(6)-Id, bla$_{CARB-2}$, sul1, tet(B)	F24:A-:B1	intI1
SRR7469870	Poultry	NA	B2	79	aac(3)-IV, aph(3'')-Ib, aph(4)-Ia, aph(6)-Id, bla$_{TEM-1A}$, dfrA5, sul1, tet(A)	F24:A-:B1	intI1
SRR7469878	Poultry	NA	G	117	aph(3'')-Ib, aph(6)-Id, dfrA5, sul1, sul2, tet(B)	F18:A-:B1	intI1
SRR7469879	Poultry	NA	G	117	bla$_{TEM-1C}$, dfrA5, sul1	F18:A-:B1	intI1
SRR7469880	Poultry	NA	A	93	bla$_{TEM-1B}$, dfrA5, tet(A)	F24:A-:B40	intI1
SRR7469887	Poultry	NA	B1	3998	aph(3')-Ia, dfrA5	F24:A-:B1	intI1
SRR7469893	Poultry	NA	D	68	aadA1, sul1, sul2	F64:A-:B27	intI1
SRR7469897	Poultry	NA	E	350	aadA1, sul2	F18:A-:B1	intI1
SRR7469899	Poultry	NA	B2	131		F24:A-:B40	intI1
SRR7469900	Poultry	NA	G	117	dfrA5, sul1, sul2, tet(B)	F18:A-:B1	intI1
SRR7469904	Poultry	NA	E	57	dfrA5, tet(A)	F2:A-:B1	intI1
SRR7469910	Poultry	NA	E	350	aac(3)-IV, aadA1, aph(3'')-Ib, aph(4)-Ia, aph(6)-Id, bla$_{TEM-1A}$, dfrA1, sul1, tet(A), tet (C)		intI1
SRR7967819	Human	NA	F	62	catA1, dfrA17	F29:A-:B10	intI1
SRR7967820	Human	NA	B2	73		F-:A-:B-	
SRR7967821	Human	NA	D	38	aadA5, aph(3'')-Ib, aph(6)-Id, bla$_{CTX-M-27}$, dfrA17, mph(A), sul1, tet(A)	F100:A-:B10	intI1
SRR7967827	Human	NA	B2	73	aadA1, aph(3'')-Ib, aph(6)-Id, bla$_{TEM-1B}$, dfrA1, sul1, sul2	F1:A1:B20	intI1
SRR7967830	Human	NA	F	62	aadA1, dfrA1, gyrA, tet(A)	F29:A-:B10	intI1
SRR7967833	Human	NA	B2	95		F29:A-:B10	
SRR7967835	Human	NA	D	349		F-:A-:B-	
SRR7967838	Human	NA	B2	95	bla$_{TEM-1B}$	F18:A-:B1	
SRR7967839	Human	NA	B2	131	aadA1, bla$_{TEM-1B}$, dfrA17, gyrA, mph(A), sul1	F2:A1:B-	intI1
SRR7967840	Human	NA	B2	95	aph(3'')-Ib, aph(6)-Id, gyrA, tet(B)	F24:A-:B1	
SRR7967841	Human	NA	B2	95	aadA2, bla$_{TEM-1B}$, catA1, dfrA12, mph(A), sul1, tet(B)	F1:A1:B23	intI1

SRR7967843	Human	NA	B2	131	*aadA5, aph(3'')-Ib, aph(6)-Id, dfrA17, gyrA, mph(A), sul1, sul2, tet(A)*	F29:A-:B10	*intI1*
SRR7967846	Human	NA	B2	12	*aadA1*, bla_{TEM-1B}, *dfrA1, sul1, tet(B)*	F-:A1:B23	*intI1*
SRR7967847	Human	NA	B2	95	*dfrA5*	F2:A-:B1	*intI1*
SRR7967850	Human	NA	B2	95	*aadA1, aadA2, cmlA1, dfrA12, mef(B), sul3*	F18:A-:B1	*intI1*
SRR7967851	Human	NA	B2	1193	*aph(3'')-Ib, aph(6)-Id*, bla_{TEM-1B}, *dfrA17, gyrA, sul2, tet(B)*	F-:A1:B10	*intI1*
SRR7967853	Human	NA	B2	5242		F-:A-:B-	
SRR7967855	Human	NA	B2	73	*aadA1*, bla_{TEM-1B}, *sul1*	F51:A-:B10	*intI1*
SRR7967856	Human	NA	B2	73		F29:A-:B10	
SRR7967857	Human	NA	B2	73	bla_{TEM-1B}	F2:A-:B-	
SRR7967881	Human	NA	B2	131	*aadA5*, bla_{TEM-1B}, *dfrA17, mph(A), sul1*	F22:A1:B20	*intI1*
SRR7967882	Human	NA	B2	131	*catA1, dfrA14, mph(A), tet(B)*	F1:A1:B1	*intI1*
SRR7967884	Human	NA	B2	73	*aph(3')-Ia, aph(3'')-Ib, aph(6)-Id, dfrA17, gyrA, mph(A), sul2, tet(B)*	F2:A-:B10	*intI1*
SRR7967885	Human	NA	B2	131	*aac(3)-IIa, aac(6')-Ib-cr, aadA5,aph(3'')-Ib, aph(6)-Id*, $bla_{CTX-M-15}$, bla_{OXA-1}, bla_{TEM-1C}, *catB3, floR, dfrA17, gyrA, mph(A), sul1, sul2*	F31:A4:B1	*intI1*
SRR7967890	Human	NA	D	38	*aac(3)-IIa, aac(3)-IId, aadA5, ampC, aph(3'')-Ib, aph(6)-Id*, $bla_{CTX-M-15}$, bla_{TEM-1B}, *dfrA17, gyrA, mph(A), sul1, sul2, tet(A)*	F29:A-:B34	*intI1*
SRR7967895	Human	NA	B2	131	*aadA5, aph(3'')-Ib, aph(6)-Id*, $bla_{CTX-M-27}$, *dfrA17, gyrA, mph(A), sul1, sul2, tet(A)*	F1:A2:B20	*intI1*
SRR7967896	Human	NA	A	10	*aadA1*, bla_{OXA-1}, *catA1, gyrA, tet(B)*	F2:A-:B-	*intI1*
SRR7967897	Human	NA	B2	73	*aadA1*, bla_{SHV-1}, $bla_{SHV-102}$, bla_{SHV-48}, *gyrA, sul1*	F51:A-:B10	*intI1*
SRR7967898	Human	NA	D	393	*aadA5*, $bla_{CTX-M-15}$, *dfrA17, erm(B), gyrA, mph(A), sul1*	F-:A1:B32	*intI1*
SRR7967899	Human	NA	B2	95	*tet(B)*	F24:A-:B-	
SRR7967900	Human	NA	B1	164		F-:A-:B-	
SRR7967901	Human	NA	B1	127	*aadA1*, bla_{TEM-34}, *sul1*	F51:A-:B10	*intI1*
SRR7967903	Human	NA	D	69	bla_{TEM-1B}, *sul1*	F29:A-:B10	
SRR7967906	Human	NA	D	349	*aph(3'')-Ib, aph(6)-Id*, bla_{TEM-1B}, *catA1, dfrA7, sul1, sul2, tet(A)*	F4:A-:B-	*intI1*
SRR7967907	Human	NA	B2	131	bla_{TEM-1B}, *catA1, dfrA7, sul1, tet(B)*	F1:A1:B23	*intI1*
SRR7967908	Human	NA	D	549	bla_{TEM-1B}, *dfrA5*	F-:A-:B1	*intI1*
SRR7967909	Human	NA	B2	73	bla_{TEM-1B}, *sul2*	F29:A-:B10	
SRR7967911	Human	NA	D	69	bla_{TEM-1B}, *sul2*	F-:A-:B1	*intI1*
SRR7967912	Human	NA	F	648	*gyrA*	F-:A-:B-	
SRR7967919	Human	NA	B2	73	*aadA1*, $bla_{TEM-141}$, bla_{TEM-1B}, $bla_{TEM-206}$, $bla_{TEM-213}$, bla_{TEM-33}, bla_{TEM-34}, bla_{TEM-57}, *sul1*	F51:A-:B10	*intI1*
